# A simple UiO-66-NH_2_@MWCNTs based electrochemical sensor for the sensitive detection of metronidazole

**DOI:** 10.5599/admet.2940

**Published:** 2025-10-03

**Authors:** Noor Abd Alkhudhur Salman, Israa A. Jassem, Isam Nghaimesh Taeb

**Affiliations:** 1 Chemistry Department, College of Science, University of Thi-Qar, Thi-Qar, Iraq; 2 Biology Department, College of Education for Pure Science, University of Thi-Qar, Thi-Qar, Iraq; 3 Pathological Analyses Department, College of Science, University of Sumer, Thi-Qar, 64001, Iraq

**Keywords:** Screen printed electrode, voltammetry, chemically modified electrodes, real sample analysis

## Abstract

**Background and purpose:**

Metronidazole (MRNZ) is a highly efficacious pharmacological agent for treating protozoal infections, including trichomoniasis, giardiasis, and disorders caused by anaerobic bacteria.

**Experimental approach:**

In the present investigation, a solvothermal approach is employed to synthesize a composite of multiwalled carbon nanotubes (MWCNTs-COOH) with UiO-66-NH_2_, resulting in the formation of UiO-66-NH_2_@MWCNTs. Given the exceptional electrocatalytic characteristics of the UiO-66-NH_2_@MWCNTs nanocomposite, it was selected as the sensing material and subsequently integrated onto the surface of a bare screen-printed carbon electrode.

**Key results:**

Under optimal experimental conditions, the developed electrochemical sensor exhibited outstanding metrics of repeatability, stability, selectivity, and reproducibility for detection across an extensive concentration range, specifically from 0.5 to 350.0 μM, while achieving a limit of detection of 0.1 μM. Furthermore, the practical application of the sensor was rigorously assessed using MRNZ tablet samples and urine specimens, resulting in a commendable recovery rate of MRNZ, ranging from 97.3 to 104.0 %.

**Conclusion:**

This research elucidates a straightforward, expedited, and significant methodology for the application of UiO-66-NH_2_@MWCNTs within the domain of electrochemical sensing.

## Introduction

The disconcerting and rapid increase in bacterial resistance to conventional therapies has resulted in a significant decline in the effectiveness of a multitude of treatments that are reliant on antibiotics [1], a situation that raises considerable alarm, particularly in light of the fact that the process of introducing new pharmaceutical agents into the healthcare market is characterized by both substantial financial costs and an extensive timeline for development. As a direct consequence of this troubling trend, it is absolutely crucial that we devise and implement innovative strategies that are designed to preserve and enhance the efficacy of antibiotics that have already received regulatory approval for clinical use [2], a concept that is exemplified by the therapeutic drug monitoring (TDM) initiatives that are currently being utilized in various clinical environments to accurately measure the concentrations of antibiotics and other related pharmacological agents present within biological fluids [3]. Through the incorporation of TDM methodologies in conjunction with the critical insights provided by pharmacokinetics, it becomes possible to identify specific instances where patients may have been administered doses of the antibiotic that are excessive, which in turn allows for the optimization of the drug concentration that is required to effectively inhibit the growth and proliferation of bacterial organisms [4]. A fundamental requirement for the successful deployment of TDM practices is the creation of assays that are not only cost-effective but also user-friendly, facilitating the accurate quantification of these essential drugs in various bodily fluids. At present, the methodologies employed to conduct such assays are predominantly characterized by their high costs and labour-intensive nature, which include methods such as radioimmunoassays, high-performance liquid chromatography (HPLC), fluorescence polarization immunoassays, enzyme immunoassays, and enzyme-linked immunosorbent assays [5]. In light of these challenges, electrochemical sensors and biosensors have emerged as highly promising alternatives that possess the capability to meet the rigorous demands of TDM, as they have exhibited impressive proficiency in the monitoring of antimicrobial agents [6], as well as in the detection of antibiotics present in aquatic environments [7], various food matrices [8], and a wide array of biological specimens [9].

The progression towards the development of effective electrochemical sensors for TDM that are also economically feasible requires a careful and deliberate selection of materials, which encompasses both the electrodes and the coating layers that are utilized for the functionalization process. Within the vast selection of materials available for these applications, carbon nanomaterials, such as graphite, nanohorns, fullerenes, carbon nanotubes, graphene, carbon nanoparticles, and nanodiamonds, deserve particular attention and emphasis, as they are noted for their reproducible electrocatalytic responses, biocompatibility, and their enhanced capabilities for electron transport [10].

**Scheme 1.**
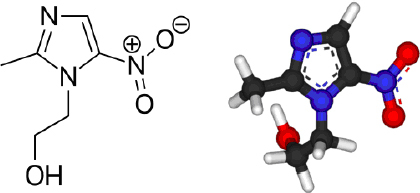
Chemical structure of MRNZ

Metronidazole (MRNZ) is classified within the category of antibiotics referred to as nitroimidazole. This pharmaceutical agent demonstrates considerable efficacy in the treatment of protozoal infections, which encompass trichomoniasis, giardiasis, and infections caused by anaerobic bacteria. The structural composition of MRNZ includes a nitro group (illustrated in Scheme 1) that undergoes reduction to a nitrosohydroxyl amino group through redox proteins that operate in anaerobic microorganisms. Consequently, MRNZ disrupts the integrity of microbial deoxyribonucleic acid (DNA) and impedes nucleic acid synthesis [11]. Given its potential carcinogenic implications, the precise quantification of MRNZ in biological matrices is imperative for safeguarding human health. A variety of methodologies have been employed to quantify MRNZ, including ion mobility spectrometry [12], high-performance liquid chromatography (HPLC) [13], colorimetry [14], fluorescence [15], and chemiluminescence [16]. Nonetheless, numerous techniques are often deemed suboptimal due to their inadequacies in affordability, accessibility, and ease of execution. In contrast, electrochemical methods present a more favourable alternative owing to their straightforward preparation, heightened sensitivity, rapid response times, cost-effectiveness, simplicity, and potential for miniaturization [17]. Furthermore, the capacity to reduce the nitro group present in MRNZ facilitates its detection via electrochemical sensing. Therefore, a variety of modified electrodes [18] have been engineered to facilitate the quantification of MRNZ.

Screen-printed electrodes (SPEs) constitute a specific category of printed electrodes fabricated through the application of the screen-printing technique. Recent electrochemical investigations have indicated that SPEs have emerged as dependable, cost-effective, portable, and pragmatic platforms for applications in electrochemical sensing [19]. It is essential to acknowledge that the use of unmodified electrodes in the electro-analytical assessment of compounds presents drawbacks, including elevated over-potential, sluggish electron transfer, constrained catalytic activity, and inadequate selectivity and sensitivity for detection purposes. The incorporation of advanced technologies, such as molecular engineering and nanotechnology, among others, can proffer innovative solutions to mitigate the limitations associated with unmodified electrodes. Nanotechnology, recognized as an emerging and progressive field, has experienced substantial advancements across various domains, including medicine, materials science, electronics, energy, and environmental stewardship [20]. Notably, recent investigations have demonstrated that nanotechnology can act as a formidable instrument in enhancing the efficacy of electrochemical sensors [21]. This technological advancement empowers researchers to devise electrochemical sensors characterized by significant attributes such as an extensive surface area, elevated porosity, and favourable electrical conductivity [22].

The material used for the electrodes represents a crucial and fundamental element within the domain of electrochemical sensors, playing an indispensable role in determining the overall efficacy and performance of the sensor in practical applications. During recent scholarly investigations, researchers and scientists have focused their efforts and intellectual resources on the progressive development of electrode materials that exhibit exceptional and remarkable physical and chemical properties, thereby enhancing their utility and functionality [23]. Within the specific context of sensor applications, metal-organic frameworks (MOFs) have garnered extensive attention for their ability to detect and identify various environmental pollutants, as documented in numerous academic studies and publications [24]. The distinctive characteristics and advantageous properties of these MOFs stem from a vast array of active sites, an increased surface area, highly porous structures, as well as their significant thermal and chemical stability and the ability to adjust pore dimensions to meet specific application requirements [25]. The prevalence of active metal sites within the structure of MOFs endows them with extraordinary electrocatalytic properties, which are highly sought after in electrochemical sensing applications [25]. Their expansive surface areas and inherent porosities not only facilitate but also expedite the rapid and efficient transport of mass and electrons toward the targeted analytes, thus enhancing the performance of the sensors [25]. However, it is imperative to acknowledge a notable limitation associated with the use of MOFs, which is their inadequate stability under various conditions [25].

Considering the elevated valence state of zirconium (Zr) and the incorporation of functional groups, particularly –NH_2_, the recently developed series of MOFs from the University of Oslo (UiO) exhibit markedly improved stability when exposed to aqueous environments, which is a significant advancement in the field. Among these, UiO-66-NH_2_, a zirconium-based metal-organic framework characterized by its amino-functionalization, is particularly well-suited for a wide range of applications in electrochemical sensing, as evidenced by various scholarly reports [26]. This specific compound features a high density of amino groups, which significantly facilitates the adsorption of analytes, including heavy metal ions, thereby enhancing its efficacy in practical applications [26]. Moreover, these amino groups serve a dual purpose, functioning as linkers for the co-immobilization of biological ligands, such as aptamers, thereby broadening the scope of their utility [26]. Despite the inherent limitation of having low electrical conductivity, MOFs can be effectively combined with highly conductive materials, including multi-walled carbon nanotubes, graphene oxide, transition metal carbides, and noble metal nanoparticles, with the objective of augmenting their electrical conductivities and overall electrochemical performance [27]. Carbon-based nanomaterials, particularly porous graphene and carbon nanotubes, are exceptionally conducive to the fabrication of MOF composites due to their ease of synthesis, superior conductivity, and cost-effectiveness, which makes them an attractive option for researchers. In particular, multiwalled carbon nanotubes (MWCNTs) have found extensive application in the research and development of electrochemical sensors, primarily due to their remarkable electrochemical performance and electrically active surfaces, thus contributing significantly to advancements in the field [28].

In this thesis, a UiO-66-NH_2_@MWCNTs composite was successfully conceptualized and synthesized, leading to the establishment of an electrochemical sensing platform (UiO-66-NH_2_@MWCNTs/SPCE) for the detection of MRNZ. The electrochemical sensing capabilities of UiO-66-NH_2_@MWCNTs/SPCE for MRNZ detection have been meticulously investigated utilizing cyclic voltammetry (CV) and differential pulse voltammetry (DPV) methodologies. Through the optimization of experimental parameters, the engineered UiO-66-NH_2_@MWCNTs/SPCE demonstrates elevated selectivity, commendable sensitivity, exceptional anti-interference properties, low detection thresholds, and stability for the identification of MRNZ. Moreover, it has been effectively applied to the analysis of real samples, resulting in satisfactory recovery rates.

## Experimental

### Chemicals and instruments

MRNZ, along with phosphoric acid, sodium hydroxide, and various other chemical reagents, was procured in an analytical grade of purity and employed for experimental purposes immediately upon receipt without any further processing. The phosphate buffer solution (PBS), which acts as the supporting electrolyte crucial for maintaining the desired pH levels during the electrochemical reactions, was meticulously prepared using phosphoric acid at a concentration of 0.1 M, and the pH was subsequently adjusted to the required level through the careful addition of an aqueous sodium hydroxide solution in a controlled manner to ensure accuracy.

To facilitate the execution of electrochemical investigations and precise measurements, which included techniques such as cyclic voltammetry (CV), differential pulse voltammetry (DPV), and chronoamperometry (CA), a PGSTAT 12N potentiostat/galvanostat/electrochemical workstation manufactured by Metrohm Autolab (The Netherlands) was utilized for its advanced features and capabilities. The screen-printed carbon electrodes (SPCEs) of type DRP-110, which were commercially available, were obtained from Metrohm-DropSens based in Oviedo, Spain, and these electrodes were specifically designed with a three-electrode configuration that includes a printed carbon working electrode (WE), a printed carbon counter electrode (CE), and a printed silver pseudo-reference electrode (RE) to ensure reliable and reproducible electrochemical measurements.

### Synthesis of UiO-66-NH_2_ MOF/MWCNTs nanostructure

The synthesis of the UiO-66-NH_2_ metal-organic framework (MOF) in conjunction with multi-walled carbon nanotubes (MWCNTs) nanostructure was initiated through the introduction of ZrCl_4_ (0.2 mmol) and 2-aminoterephthalic acid (0.2 mmol) into a beaker containing dimethylformamide (DMF) (20 mL), wherein this solution underwent magnetic stirring for approximately 5 minutes. In the subsequent phase, acetic acid (5.0 mL) was incrementally introduced to the precursor solution while maintaining continuous agitation. Following this, carboxylated MWCNTs (0.0075 g) were dispersed in the aforementioned solution and subjected to ultrasonication for approximately 45 minutes to achieve a uniform suspension. Upon completion of the ultrasonication process, the mixture was transferred to an autoclave and subjected to thermal treatment at 120 °C for 48 hours. After the 48-hour interval, the autoclave was permitted to cool to room temperature. Subsequently, the synthesized precipitates were retrieved by centrifugation of the reaction mixture. The collected precipitates were washed with DMF and methanol to remove any residual impurities. Ultimately, the purified precipitates were desiccated at 70 °C for 15 hours through vacuum drying.

### Preparation of modified SPCEs

For the preparation of UiO-66-NH_2_@MWCNTs/SPCE, 1.0 mg of the prepared UiO-66-NH_2_@MWCNTs was weighed and dispersed into 1.0 mL deionized water, followed by an ultrasonication process to obtain a homogeneous suspension (1.0 mg mL^-1^) of this nanostructure. Then, 3.0 μL of a well-dispersed suspension of UiO-66--NH_2_@MWCNTswas dropped onto the surface of the SPCE and finally dried for 15 min under ambient conditions to evaporate the solvent from the suspension. This electrode was named UiO-66-NH_2_@MWCNTs/SPCE.

### Preparation of samples

#### MRNZ sample preparation

To facilitate the preparation of a pharmaceutical sample for analytical purposes, a total of five MRNZ tablets, each containing an active ingredient dosage of 250 mg, were meticulously ground into a fine powder using a mortar and pestle, ensuring a homogeneous consistency crucial for subsequent analyses. Following this grinding process, a predetermined and precisely measured amount of the resulting homogenized powder was carefully weighed to ensure accuracy and then transferred into a 100 mL volumetric flask, which is designed to contain a specific volume of liquid. The volumetric flask was subsequently filled to the calibration mark with deionized water, which serves to eliminate any potential contaminants. The contents were then subjected to ultrasonication for a minimum duration of 20 minutes to ensure thorough mixing and dissolution of the powder. After the ultrasonication process was completed, the mixture in the flask was filtered through filter paper, which serves to remove any undissolved particulates that could interfere with later analytical measurements. In the next phase of the procedure, a specific volume of the filtered resultant sample was diluted with phosphate-buffered saline (PBS), which was prepared at a concentration of 0.1 M and adjusted to a neutral pH of 7.0, thus creating an appropriate environment for electrochemical analysis. Ultimately, both the unspiked and spiked tablet samples, which contained varying concentrations of the active pharmaceutical ingredient MRNZ, were separately transferred into an electrochemical cell, where they underwent quantitative analysis. This analysis was performed using DPV, a highly sensitive electrochemical technique, which employed the standard addition method to ensure the accuracy and reliability of the results obtained. Through these meticulous procedures, the study aimed to achieve a comprehensive understanding of the concentration levels of MRNZ present in the samples, thereby contributing valuable data to the field of pharmaceutical analysis.

#### Preparation of urine sample

The human urine specimen, which was obtained from a qualified laboratory technician, underwent a rigorous centrifugation process at an impressive rate of 6000 revolutions per minute for a standardized duration of precisely 5 minutes to ensure optimal separation of components. Following this centrifugation step, the resultant supernatant was meticulously diluted with a phosphate-buffered saline solution, specifically formulated to a concentration of 0.1 M at a neutral pH level of 7.0, in a carefully calculated ratio of 1 part urine to 5 parts saline to maintain the integrity of the sample. Subsequently, the distinct urine samples, one of which remained unspiked while the other was fortified with varying concentrations of MRNZ at 5.0, 7.0, 9.0 and 11.0 μM, were methodically transferred to an electrochemical cell designed for the precise quantitative analysis of MRNZ. This analysis was conducted utilizing a standard addition approach through the application of DPV.

## Results and discussion

### Voltammetric responses of unmodified SPCE and UiO-66-NH_2_@MWCNTs/SPCE towards 100.0 μM MRNZ

The DPV responses of the composite material UiO-66-NH_2_@MWCNTs/SPCE, when subjected to varying concentrations of 40.0 μM MRNZ, were meticulously recorded with the objective of investigating the intricate influence that pH levels, which were systematically varied from pH 2.0 to 9.0, exert within a 0.1 M PBS solution. The data obtained from the DPV responses compellingly indicated that the pH of the supporting electrolyte solution holds considerable significance, as it can dramatically affect the voltammetric response of the engineered sensor towards the target analyte MRNZ. A thorough analysis of the results from the DPV experiments elucidated that the oxidation peak potential (*E*_pa_) corresponding to MRNZ exhibited a notable decrease that occurred in tandem with an increase in the pH value when transitioning from pH 2.0 to 9.0, thereby providing substantial evidence that protons are integral to the oxidation mechanism of MRNZ taking place on the surface of the UiO-66-NH_2_@MWCNTs/SPCE.

Furthermore, the oxidation peak currents of MRNZ displayed a steady and gradual increase as the pH level transitioned from the acidic environment of pH 2.0 PBS to a more neutral pH 7.0 PBS, thereby establishing a positive correlation with the observed elevation in pH. However, a subsequent decline in the peak current (*I*_pa_) of MRNZ was distinctly observed as the pH values were increased further from 7.0 to 9.0. Consequently, through comprehensive evaluation and analysis, a 0.1 M PBS solution at the neutral pH level of 7.0 was ultimately determined to be the most optimal medium for the analytical assessment of MRNZ, ensuring the highest sensitivity and specificity in detection.

**Figure 1.**
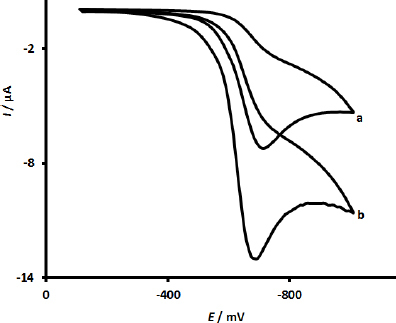
CV responses of unmodified SPCE (a) and UiO-66-NH_2_@MWCNTs/SPCE (d) towards 100.0 μM MRNZ in 0.1 M PBS (pH 7.0) (scan rate = 50 mV s^-1^)

Figure 1 illustrates the CVs that depict the comparative electrochemical performance of a variety of electrodes, specifically the unmodified SPCE (designated as a) and the modified UiO-66-NH_2_@MWCNTs/SPCE (designated as b), in relation to the redox reaction characteristics of MRNZ.

In the case of the unmodified SPCE, MRNZ demonstrated a relatively inadequate electrochemical response, as clearly depicted in cyclic voltammogram a, indicating that the electron transfer kinetics during the reduction process of MRNZ on this unmodified electrode were notably sluggish and inefficient. Furthermore, it was observed that markedly higher detection efficiency for MRNZ was accomplished through the utilization of UiO-66-NH_2_@MWCNTs in the modification of SPCE, as evidenced by the data presented in cyclic voltammogram b. At the interface of the UiO-66-NH_2_@MWCNTs/SPCE, a pronounced reduction in the reduction peak potentials was observed, which coincided with an even more substantial increase in the reduction peak currents when compared to those observed at the unmodified SPCE. This exceptional performance of the modified sensor can be attributed to the remarkable characteristics inherent in Ni-UiO-66-NH_2_ and MWCNTs, which include their extensive surface areas and the superior electrical conductivity of MWCNTs, in conjunction with their synergistic effects that serve to enhance the electrochemical reduction processes associated with MRNZ significantly.

### Influence of potential scan rate

**Figure 2.**
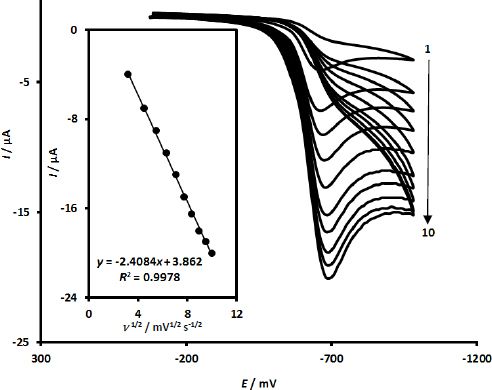
Cyclic voltammetric responses of UiO-66-NH_2_@MWCNTs/SPCE for a concentration of 100.0 μM MRNZ in 0.1 M phosphate-buffered saline (PBS) at a pH of 7.0 across various scan rates (numbers 1-10 correspond to: 10, 20, 30, 40, 50, 60, 70, 80, 90 and 100 mV s^-1^). The associated linear representations of cathodic peak current (*I*_pc_) plotted against *ν*^1/2^ (Inset)

The intricate and multifaceted relationship that exists between the reduction peak currents and the potential scan rate throughout the reduction process of the compound MRNZ was meticulously examined by employing CV, which involved a systematic alteration of the scan rate (*ν*) across a defined range, specifically from 10 to 100 mV s^-1^. The cyclic voltammograms that correspond to the advanced sensing platform comprising UiO-66-NH_2_@MWCNTs/SPCE were meticulously acquired in a 0.1 M PBS maintained at a neutral pH of 7.0, with MRNZ at a concentration of 100.0 μM under varying scanning rates, as is clearly depicted in Figure 2.

As is further elucidated in the inset of Figure 2, the reduction peak currents, which are associated with MRNZ, reveal a pronounced linear increase when considered in relation to the square root of the scan rate (*ν*^1/2^), a finding of considerable significance. This particular observation strongly suggests that the behaviour observed is not merely coincidental but rather indicative of a diffusion-controlled system, a conclusion that has implications for our understanding of the underlying mechanisms governing the reduction processes involved. The systematic approach taken in altering the scan rates enables a comprehensive analysis of the reduction characteristics, providing valuable insights into the electrochemical behaviour of MRNZ. The results obtained from this investigation underscore the intricate interactions that occur within the electrochemical environment, particularly under varying conditions, which can significantly influence the efficacy of the sensing platform. The findings contribute to the broader discourse on reduction chemistry and its applications in sensor technology, thereby enhancing our understanding of the fundamental principles at play. Overall, the research not only elucidates the specific behaviour of MRNZ but also enriches the field of electrochemistry with empirical data that could inform future studies and applications.

### Chronoamperometric studies

**Figure 3:**
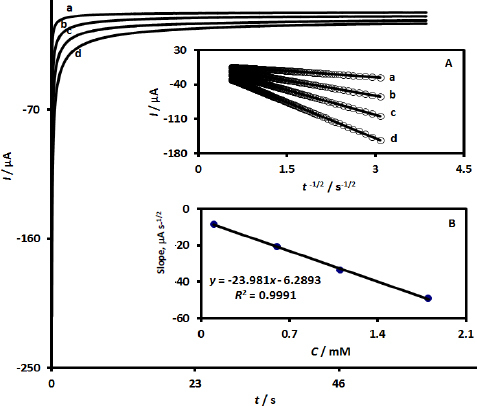
Chronoamperometric responses of UiO-66-NH_2_@MWCNTs/SPCE in PBS (0.1 M at pH: 7.0) containing MRNZ at differing concentrations: 0.1, 0.6, 1.1 and 1.8 mM (chronoamperograms labeled from a to d). Linear plots depicting current against *t*^-1/2^ derived from chronoamperometric data at varying MRNZ concentrations (Inset A), along with a linear plot of the slopes obtained from Inset (A) against MRNZ concentration (Inset B)

The chronoamperometric measurements of UiO-66-NH_2_@MWCNTs/SPCE at an applied potential of -0.71 V in a 0.1 M PBS solution (pH 7.0) containing varying concentrations of MRNZ were documented to evaluate the diffusion coefficient of MRNZ (Figure 3). As depicted in Figure 3A, a significant linear relationship is evident between the current (*I*) and the inverse of the square root of time (*t*^-1/2^).

These linear representations, which are derived from the chronoamperograms obtained for each concentration of MRNZ over a specified time interval, are termed Cottrell plots. Additionally, a linear correlation was identified between the slopes of the Cottrell plots and the MRNZ concentrations (Figure 3B). Therefore, the diffusion coefficient of MRNZ can be computed as defined by the Cottrell equation (*I* = *nAFCD*^1/2^π^-1/2^*t*^-1/2^). In this equation, *I* represent the peak current, *n* denotes the number of electrons transferred, *F* is Faraday's constant, *A* indicates the geometric surface area of the working electrode, *C* refers to the concentration of the analyte, *D* symbolizes the diffusion coefficient, and υ pertains to the scan rate. By employing the slope from the linear plot in inset B in conjunction with the Cottrell equation, the average diffusion coefficient of MRNZ was calculated to be 1.23×10^-5^ cm^2^ s^-1^.

### Quantitative determination of MRNZ using differential pulse voltammetry method

**Figure 4.**
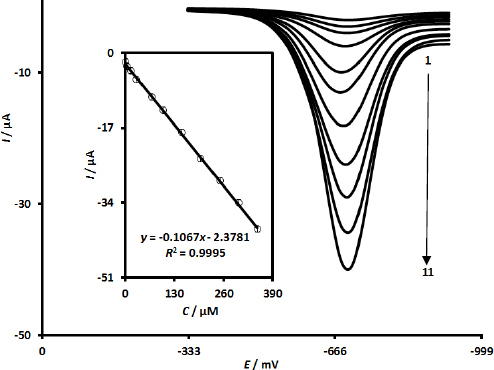
DPV responses of UiO-66-NH_2_@MWCNTs /SPCE towards MRNZ determination at various concentrations: numbers 1-11 correspond to 0.5, 5.0, 15.0, 30.0, 70.0, 100.0, 150.0, 200.0, 250.0, 300.0 and 350.0 μM in PBS (0.1 M at pH 7.0). Calibration plot of MRNZ (Inset)

The DPV technique was employed to conduct quantitative assessments of MRNZ. The DPV responses of the UiO-66-NH_2_@MWCNTs/SPCE for the detection of various MRNZ concentrations are illustrated in Figure 4. It is observed that the peak current (*I*_pa_) of the recorded voltammograms exhibited a progressive increase corresponding to the rising concentrations of MRNZ. Moreover, as demonstrated in the inset of Figure 4 (calibration graph for MRNZ quantification at the modified SPCE), the oxidation peak current of MRNZ showed a linear relationship with its concentration, ranging from 0.5 to 350.0 μM. The limit of detection (LOD) was calculated to be 0.1 μM at a signal-to-noise ratio of 3. Furthermore, the DPV analysis indicates that the UiO-66-NH_2_@MWCNTs/SPCE sensing platform exhibits a notable sensitivity of -0.1067 μA μM^-1^ for the quantification of MRNZ.

### Repeatability and stability studies

The reproducibility and durability of the UiO-66-NH_2_@MWCNTs/SPCE sensing platform for the quantification of MRNZ were assessed through the DPV technique. To assess reproducibility, ten consecutive measurements of a 35.0 μM MRNZ solution prepared in 0.1 M PBS were conducted utilizing the same electrode. These measurements exhibited similar current responses, yielding a relative standard deviation (RSD) of 4.1 %. This finding indicates that the UiO-66-NH_2_@MWCNTs/SPCE exhibits noteworthy reproducibility. To assess its durability, the UiO-66-NH_2_@MWCNTs/SPCE sensing platform was constructed and maintained under standard ambient conditions, with DPV assessments of 35.0 μM MRNZ conducted weekly over a period of three weeks. Over this duration, the current response demonstrated a slight reduction, achieving 95.9 % of the initial value after three weeks. This minimal decline implies that the stability of the developed sensing platform is likewise satisfactory.

### Application of UiO-66-NH_2_@MWCNTs /SPCE for analysis of samples

The UiO-66-NH_2_@MWCNTs/SPCE sensor framework was ultimately utilized to quantify MRNZ in both MRNZ tablet formulations and urine samples. Initially, the prepared specimens were placed into the electrochemical cell for DPV evaluations without incorporating standard MRNZ solutions. Subsequently, known concentrations obtained from standard MRNZ solutions were integrated into the prepared specimens, leading to the generation of a series of samples. Thereafter, DPV examinations of these samples were performed, and the respective concentrations of each sample were inferred based on the established calibration curves. The results from this investigation are delineated in Table 1.

**Table 1.** The summarized results from the analysis of MRNZ in MRNZ tablet and urine samples using UiO-66-NH_2_@MWCNTs/SPCE sensor

Sample	Spiked concentration, μM	Found concentration, μM	Recovery, %	RSD, %
MNZ tablet	0	5.1	-	2.9
	2.0	7.0	98.6	3.6
	4.0	9.4	103.3	2.1
	6.0	11.2	100.9	1.7
	8.0	13.0	99.2	2.8
Urine	0	0	0	0
	5.0	5.1	102.0	3.3
	7.5	7.3	97.3	2.5
	10.0	10.4	104.0	2.2
	12.5	12.4	99.2	1.9

It is apparent from Table 1 that the recoveries of MRNZ varied from 97.3 to 104.0 %, while the relative standard deviations (RSDs) remained below 3.6 %.

## Conclusion

In conclusion, the UiO-66-NH_2_@MWCNTs composite was synthesized and demonstrated significantly enhanced catalytic activity attributable to its unique structural characteristics and the synergistic interactions between UiO-66-NH_2_ and MWCNTs. The analytical performance of the constructed sensor for the sensitive and selective detection of MRNZ was effectively validated through both controlled laboratory solutions and analyses of real-world samples. Under optimized experimental conditions, the detection limit was ascertained to be 0.1 μM, the quantification limit was established at 0.5 μM, and the sensitivity was quantified at -10.67 μA μM^−1^. The primary advantage of the developed sensor resides in its straightforward and economically viable preparation of the electrode modifier, coupled with its high sensitivity, selectivity, and reproducibility. These results indicate that the UiO-66-NH_2_@MWCNTs/SPCE sensor possesses the potential to be advanced into a simple, rapid, practical, and effective electrochemical platform for applications in MRNZ analysis.
